# Association of Gastroesophageal Reflux Disease With Dental Erosion

**DOI:** 10.7759/cureus.30381

**Published:** 2022-10-17

**Authors:** Ananya Chakraborty, Ashish P Anjankar

**Affiliations:** 1 Gastroenterology, Jawaharlal Nehru Medical College, Datta Meghe Institute of Medical Sciences, Wardha, IND; 2 Biochemistry, Jawaharlal Nehru Medical College, Datta Meghe Institute of Medical Sciences, Wardha, IND

**Keywords:** damage, pepsin, gastric acid, dental erosion, gerd, gastro-esophageal reflux disease

## Abstract

Gastroesophageal reflux disorder (GERD) or chronic acid reflux disorder is a condition in which acid-containing contents continuously leak from the stomach and return to the esophagus. Acid reflux disease occurs in nearly every person at some unspecified time. In reality, it is considered as a reoccurrence of acid reflux disease disorder and heartburn, every day. However, when you have acid reflux disorder/heartburn greater than two times every week over numerous weeks, constantly take heartburn medicinal tablets and antacids. However, if your signs and symptoms and symptoms keep returning, you can have superior GERD. Your GERD needs to be handled with the aid of your healthcare employer. Now not simply to alleviate your symptoms, but because of the reality, GERD can result in extra intense issues. Dental erosion (DE) is the shortage of the ground of your tooth because of acids you eat or drink or acids arising from your stomach. Those acids can wash away the tough substance that makes up your enamel, number one to tooth floor loss. Acid also can melt the teeth floor, making it much less complicated to wear away with the beneficial aid of erosion. This is called acid put on or erosive enamel wear. The belly contains many sturdy acids that are used to digest food. Vomiting and reflux can reason those belly acids to enter your mouth. Gastric acids are very sturdy and might purpose considerable harm to the tooth. DE is the lack of the enamel's hard tissues due to the interplay of gastric juice, pepsin, and acid.

## Introduction and background

Dental erosion (DE) is described as the loss of hard dental tissue caused by a chemical reaction without the presence of bacteria [1]. The upper incisors' palatal surfaces are first attacked by refluxed acid; if the situation persists, the posterior teeth in both arches' occlusal surfaces erode in the secondary stage. Only when acid reflux lasts for a long time can the labial or buccal surfaces experience erosion. Because the tongue is constantly in contact with gastric acid and the major salivary glands protect the palatal surfaces of the maxillary teeth, they are the first to be impacted. According to research, the grading of DE depends on how forcefully the reflux exits the pharynx and later enters the mouth. The lower teeth are initially shielded by the tongue, but if the disease persists, the occlusal and buccal surfaces of these teeth will deteriorate [2]. The time period "teeth wear" encompasses attrition, abrasion, and erosion and does now not specify the mode of damage, bearing in mind a blended etiology. For effective control, the etiology should be decided. The topics of recognized hazard factors for attrition, abrasion, and erosion are included. The prognosis is made more significantly hard by using many etiologies, which may additionally adjust the scientific look or pattern of tooth attrition [3].

DE and gastroesophageal reflux disorder (GERD) may be related, but causality or a temporal relationship has no longer been mounted. Moreover, it became hard to make conclusive findings due to the dearth of studies, the small pattern sizes, and the variety of disease diagnoses. The general public of GERD therapy trials on youngsters did not display any development in extra-oesophageal signs [4].

DEs have been discovered in 76.7% of individuals inside an experimental group and 53.3% of patients in the control institution. The lower first molars and the enamel within the upper jaw's front place had the most acceptable common values for the DE index. DEs can be the extraesophageal signal of GERD [5].

## Review

Only seven studies out of 32 studies found a significant link between GERD patients and teeth degradation. This comprehensive analysis led to a connection between oral disorders and GERD (DE and halitosis). Different indicators were employed in the epidemiological surveys to investigate GERD and DE [6].

While dental apatite is subjected to non-bacterial acids, DE effects by its dissolution, GERD, brought on with the aid of continual regurgitation of stomach contents into the oropharynx, is one of the risk factors for enamel erosion. Accordingly, GERD sufferers may also have erosive enamel lesions and different extraesophageal signs and symptoms. Erosive dental lesions are more common in this group, and GERD may have seemed a hazardous aspect of their prevalence. However, several additional elements, together with nutritional practices, way of lifestyle, abrasion, and bruxism, are frequently linked to the superiority and tooth erosion severity among the population of the world. Numerous discomforts and extraesophageal signs, which include mucous lesions and enamel erosion, may be introduced via GERD. When uncovered to non-bacterial acids, DE is a highly not unusual disorder marked by the irreversible loss of dental structure. DE, which impacts each deciduous and permanent tooth, has a complicated cause [7].

It has been suggested that DE is an extraesophageal symptom of GER illness. Between April 1998 and May 2000, a prospective study with 253 people was conducted. One hundred eighty-one sufferers with GER sickness and 72 healthy volunteers were divided into two observation organizations. All individuals underwent a clinical evaluation that included measuring their body mass index, smoking and drinking habits, periodontal and dental, and an exam with a dentist unaware of the subjects' conditions. Clinical indicators were comparable between the two groups [8].

GERD is described as the decreased esophageal sphincter's involuntary muscle relaxation, which enables refluxed acid to journey upward through the esophagus and into the mouth cavity. Six typical symptoms, such as enamel erosion, chronic cough, hoarseness, and noncardiac chest ache, are regularly overlooked and poorly understood. Because most patients are unaware that they have the disorder, dentists often make the initial analysis of GERD through tooth erosion. Belching and heartburn are two common GERD signs and symptoms, while some people do not have any signs at all. Dentists ought to test for GERD in their patients [9]. On esophageal pH monitoring, 10 out of the 12 patients in the dental group showed GER. Seven had proximal reflux, and nine had distal reflux Only one person experienced reflux while supine, whereas two experienced reflux in upright and supine positions [10].

In GERD, esophagitis and supraesophageal symptoms emerge from reflux that happens more frequently and for a more extended period than is natural. The material of the teeth interacts with the acid, which may even enter the mouth, and demineralization, a type of DE, occurs. Clinical research has also shown that patients with GERD have teeth erosion far more frequently than patients without the condition. It was also confirmed that GERD is more common in people with idiopathic tooth erosion than in the "uneroded" group. These findings imply that a gastroenterological condition, which may not yet have been identified, should be considered by the dentist in cases of prolonged tooth erosion [11].

DE is an incurable lesion that could indicate a medical condition. The propelling of stomach contents into the mouth is essentially the mechanism of internal erosion. Its miles regarded that common regurgitation or vomiting is terrible for the tooth because the belly's acidity may additionally fall underneath pH 1. The enamel of the tooth possesses an extreme pH of 5.5. Acidic compounds with a pH below 3.7 have produced apparent erosion in vitro [12].

The hydroxyapatite crystals in the enamel of the tooth can be dissolved through any acid with a pH of less than 5. The pH of gastric reflux is much less than 2.0. One of the GERD signs that may occur out of doors of the esophagus is DE, following research. Exclusive grading scales may be used to describe the severity of erosion damage. The quantity of exposed dentin is taken under consideration while scaling teeth. Early tooth decay reveals a loss of visible vibrancy and floor elements. The tooth has an ugly yellowish tint because of tooth thinning. The occlusal and palatal surfaces of the maxilla are most frequently affected by erosion. On the cusp, hints of the molar teeth and cupping lesions can be observed. The tooth can be overly touchy to stress, sweetness, temperature, and other sensations. Lack of vertical size, immoderate closure, and bite crumble are possible side results of the system as it develops. If bruxism attrition happens on top of abrasion, the loss of tooth structure can be multiplied. Additionally, those with GERD can enjoy erythema of the soft palate and uvula. While the standard signs of heartburn, bitter flavor or belching do not occur, GERD is said to be silent. Primarily based on the styles of erosion, dentists might be the primary ones to speculate approximately GERD. DE ought to affect up to 20% of human beings with GERD. In keeping with estimates, sufferers with negative enervated salivary float have a 5 times better hazard of developing erosion than people with a regular flow. [13]. When in comparison to wholesome members, kids affected by GER ailment had a greater chance of growing DE; among GERD children, DE becomes connected to the use of adult toothpaste [14].

A multidisciplinary strategy involving a family doctor, dentist, prosthodontist, and gastroenterologist is used to treat DE brought on by GERD. DE should, wherever possible, be treated with the least amount of intervention possible, and this treatment should include biomimetic materials, adhesive restorations, remineralization, microflora management, and remineralization. The flow of stomach contents into the esophagus is known as GER, and the symptoms or side effects of GER are known as GERD. The diagnosis of GERD benefits from 24-hour esophageal pH monitoring [15].

DE was observed to be more common in GERD patients than in those with suspected GERD or in healthy people. According to this study, in a sample of the Iranian population, people with GERD have a higher risk of having DE than people in good health [16].

The teeth may completely disintegrate due to the deterioration of the outer enamel of the tooth caused by crystal breakdown. Because of how the tongue rests, the palatal and occlusal surfaces of the upper enamel and occlusal/lingual and buccal surfaces of the decreased enamel are where this harm most often occurs in GERD sufferers [17]. Table 1 depicts the reflux total and upright action in the group either affected by GER ailment or not [18].

**Table 1:** Different refluxes in people with or without GERD. In patients with and without GERD, the total median percentage of time the pH dropped less than 4 throughout the twenty-four-hour pH measurement, as well as the percentage of time that the patient had either total reflux or upright reflux, was recorded [18].

	The affected person with GastroEsophageal Reflux ailment.	The person who is not affected with GastroEsophageal Reflux ailment.
Total Reflux	11.6	1.4
Upright Reflux	12.1	1.5

Ten subjects had GERD diagnoses, while another 10 had manometry results that fell below the threshold for GERD. In general, GERD patients scored considerably higher on the TWI compared to control patients. All quadrants, except for the region of the anterior mandible, in which no change was observed, showed greater TWI scores in GERD participants. The findings suggested a connection between the prevalence of GERD in certain participants and dental structural loss, as determined by the TWI index [19].

In adults, the grade of esophageal or buccal subjection to pH of low value appears to be associated with the existence of GERD symptoms and the severity of DE. In patients with GERD, examining the mouth to look for DEs should become a routine procedure [20]. Thirty-seven children who underwent elective endoscopy for potential GERD had their teeth examined and GERD affected 24 children. These 24 children experienced GERD and DEs. The posterior teeth were more involved in the erosion patterns [21]. Particularly in individuals without GERD, taurocholic acid predominated in the salivary bile. The enamel structure was not negatively impacted by this bile acid [22]. Due to their cosmetic and functional effects on the stomatognathic apparatus, extra-esophageal manifestations that influence the oral structures are particularly significant. The authors discuss a case of GERD that was recently diagnosed and successfully treated along with significant cosmetic lesions of the front teeth [23].

Dentists may be the first to suspect GERD in these “silent refluxers,” especially when they notice inexplicable cases of tooth erosion that may also be accompanied by hypo salivation. There is a definite, albeit variable, link between GERD and tooth erosion, according to a large number of laboratory research in adults and children, mostly case-control and observational clinical studies [24]. Table 2 shows the pattern of erosion in the control group and patients affected by GERD [25].

**Table 2:** Different levels of erosion of teeth in patients with GERD and control group. Contrasting of Dental Erosion pattern in gastroesophageal reflux disease GERD and control groups [25].

		Pattern of erosion	Pattern of erosion	Pattern of erosion
	Presence of Dental Erosion	Localized	Generalized	Both
Patient with known GERD	53	18	34	1
Control	11	5	6	0

In the adult population, DE and GERD were strongly associated, but the relationship in the juvenile group was shown to be less significant. Through lifestyle modifications and medication, early detection and remedy of refluxed acid in both age categories can stop future decay and tooth loss [26]. With silent GERD, the combination of acid erosion and attrition erosion can result in significant tooth surface loss [27]. Long-term acid exposure to teeth causes the surface of the teeth to dissolve. At pH values of 5.5 and lower, enamel dissolves. Acid from intrinsic, extrinsic, or a combination of intrinsic and extrinsic sources, as well as other sources, is what causes DEs. Regurgitation, vomiting, and GERD are included in sources that are intrinsic to oral [28].

DE is more common in GERD patients than in healthy individuals. The study's clinical implications are that all GERD patients must be checked for tooth erosion and that these patients must be properly educated about the harmful effects of GERD on dental tissues. To observe the prevalence and prevalence of teeth erosion in the GERD population, in addition, studies with a larger sample size are needed [29].

The connection between adult caries and reflux was examined in three trials. Five research have examined the role of reflux in the emergence of periodontal and gingival diseases [30]. According to the results of the current study, GERD children had a higher risk of tooth erosion and caries than healthy children [31].

Dental deterioration and unaroused salivary flow rate and other outcomes have been related. Reduced salivary flow rates are significantly correlated with oral dietary acid clearance and buffering ability [32]. Oral symptoms of GERD are probably linked to swallowing difficulties or reduced salivary flow volume. Patients with GERD may additionally benefit from a remedy for mouth dryness delivered by way of decreased salivary glide volume in addition to swallowing feature rehabilitation [33].

According to the fixed effects model, there was a significant correlation between the presence of DE and GERD when compared to controls. Additionally, it was discovered that DE and GERD were significantly related [34]. DE and GERD had a positive correlation. Consuming carbonated beverages can raise your risk of GERD and DE [35]. The patient's primary symptom is upper incisor palatal erosion. In GERD patients who also have symptoms in their airways, acid reflux is the main contributing factor to DE [36]. Patients with GERD had higher rates of DE, particularly on the palatal and lingual tooth surfaces [37]. DE was identified as an extraesophageal GERD manifestation in studies that found a substantial association between GERD and DE [38].

## Conclusions

There is enough evidence in the form of research articles that indicates a significant association between GERD and DE. Although observed more in children, DE is a detrimental process that has an important underlying etiology in any age group. A large proportion of patients diagnosed with DE had had a history of GERD; however, a lesser proportion of people who did not suffer from GERD developed DE. GERD is one of the leading causes of DE. Treatment of the gastric secretion buffer system can reduce the occurrence of erosion of the tooth quite prominently, if not entirely.
